# Fabrication of *in situ* self-assembled MOF-on-MOF heterostructures and incorporation of biosynthesized CQD nanomaterial

**DOI:** 10.1039/d6ra05669e

**Published:** 2026-09-07

**Authors:** Aysenur Aygun, Damla Ikballi, Meliha Koldemir Gunduz, Gullu Kaymak, Elif Aydın, Fatih Sen

**Affiliations:** a Department of Biochemistry, Sen Research Group, Kutahya Dumlupinar University 43000 Kutahya Turkiye fatihsen1980@gmail.com; b Department of Engineering Basic Sciences, Faculty of Engineering and Natural Sciences, Kutahya Health Sciences University Kutahya Turkiye; c Training and Research Center, Kutahya Health Sciences University 43100 Kutahya Turkiye; d Department of Medical Microbiology, Faculty of Medicine, Agrı İbrahim Çeçen University Agrı Turkiye

## Abstract

With the rapid development of industry, organic dye pollution and heavy metal ion contamination pose a major threat to water safety. Furthermore, bacterial resistance and the difficulty of cancer treatments are among the leading problems encountered in the health sector. There is a growing need for fast and effective multifunctional nanomaterials to both prevent environmental pollution and address health problems. Carbon quantum dots (CQDs), with their high photoluminescence, low toxicity, and tunable surface chemistry, have emerged as versatile nanomaterials in sensing technologies. Additionally, metal–organic lattice structures (MOFs), with their high surface areas and tunable porous structures, possess tunable functionalities for molecular recognition and analyte enrichment. The synergistic integration of CQDs and MOFs in the development of multifunctional hybrid materials is a current research topic. In this study, CQDs obtained from banana peels, a common carbon-rich waste product, were combined into a hybrid structure with a MOF-on-MOF architecture to improve their optoelectronic properties and create synergistic interactions. The chemical structure of the synthesized composite material was determined using FTIR analysis, while properties such as surface morphology and average particle size were analysed using FE-SEM and TEM analyses, and crystal structure by XRD analysis. This composite structure has been used in studies on fluorescence sensor detection for heavy metal ion detection. In addition to fluorescence sensor studies, this composite structure has also been used in photocatalytic studies on the removal of azo-toxic dyes such as methylene blue (MB) from wastewater, in antibacterial studies, and in anticancer studies. After 150 minutes against MB, 91.63% photocatalytic activity was observed. The LOD value for Cr^6+^ detection in the hybrid material was calculated as 3.28 µM. The results obtained demonstrate that the developed CQD@MOF-on-MOF hybrid structure is a multifunctional nanoplatform exhibiting high performance in Cr^6+^ detection as a fluorescent sensor, organic dye removal through photocatalytic activity, and antibacterial and anticancer applications. This study shows that the hybrid material obtained with the MOF-on-MOF structure of CQDs synthesized from sustainable biomass sources is promising for environmental and biomedical applications.

## Abstract 2

CQDs were integrated into a hybrid MOF-on-MOF architecture. This composite was used as a fluorescent sensor for Cr^6+^ detection. Furthermore, their photocatalytic activity against biological and MB dyes was investigated.

## Graphical abstract

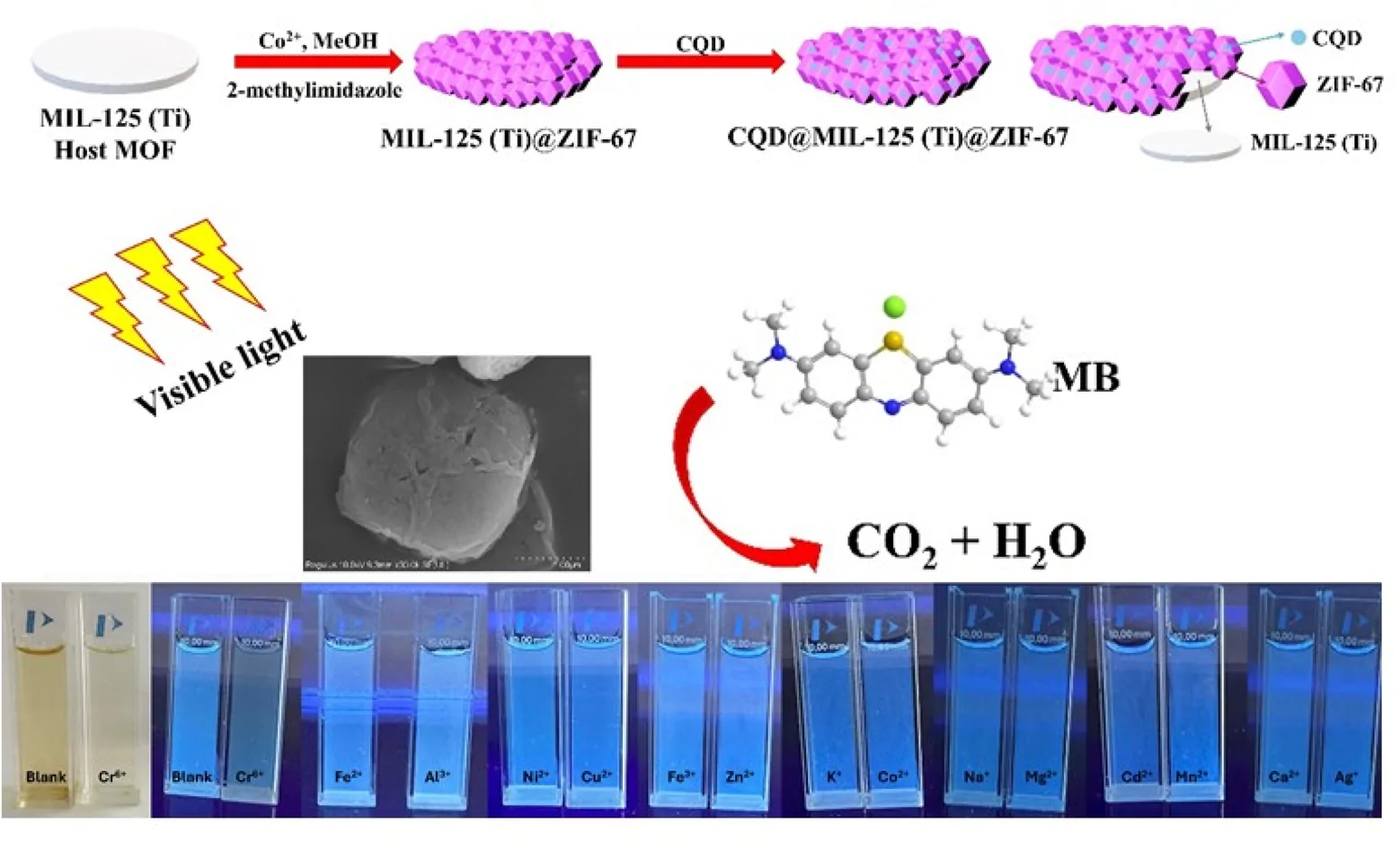

## 1. Introduction

The discharge of industrial wastewater is one of the most important causes of water pollution. Synthetic dyes in wastewater are persistent due to their chemical stability and are toxic to living organisms.^1^ Traditional methods exist for dye removal from wastewater. However, although these methods have advantages, their use is limited due to disadvantages such as high operating costs, complex operating conditions, secondary pollution, and incomplete degradation.^2^ Therefore, photocatalysts are emerging as an alternative to traditional methods. Furthermore, heavy metals are another pollutant encountered in wastewater. Heavy metal ions pose environmental and biological risks because they cause high toxicity in living organisms.^3^ This necessitates monitoring the heavy metal content of wastewater with sensors. The use of fluorescence-based sensors is noteworthy in monitoring heavy metals in wastewater due to their high sensitivity, on-site analysis, and fast response times.^3^

Carbon quantum dots (CQDs) are widely used in the fluorescence detection of metal ions due to their low toxicity, high solubility in water, strong fluorescence properties, and ease of synthesis.^3,4^ CQDs can be efficiently synthesized from various natural waste sources such as fruit peels (banana, lemon, orange), and other biomass residues, allowing for low-cost production.^3^

Metal–organic frameworks (MOFs) are structures composed of metal ions or clusters and organic ligands that bind them together. MOFs have been quite popular in recent years due to their unique chemical properties, exceptional number of micro-mesopores, and high surface area.^5^ Therefore, MOF-based materials are used in a wide range of applications such as sensors, catalysts, photocatalysts, drug delivery, and energy storage.^6^

Recently, carbon quantum dots (CQDs) and metal–organic frameworks (MOFs) have shown significant potential for sustainable, environmentally friendly, and biological applications.^7^ MOF-on-MOF heterostructures, modern structures formed by the interaction of these materials, are attracting great interest, particularly in fields such as bioengineering, catalysis, sensing, and photocatalysis.^8–15^ Metal–organic frameworks, thanks to their regular structures, porosity, and high surface area, play an important role in environmental applications such as the detection of heavy metals (Cr^6+^*etc.*) and the removal of organic pollutants. The photocatalytic activity of these types of materials can significantly prevent the release of toxic compounds (such as Cr^6+^) and organic pollutants (methylene blue dyes, *etc.*) into the environment, offering a major advantage for environmental protection and water treatment.^16–18^ The high solubility of CQDs in water and their high photochemical activity have led to the widespread use of such materials in fields like environmental monitoring and biological sensing. Detecting environmental pollutants such as oxidation and nitration using CQD-based sensors is emerging as an effective solution for environmental monitoring. MOFs, with their high surface area and pore structure, provide a support structure for CQDs, enabling high sensitivity, selectivity, stability, and rapid detection. The interaction between CQDs and MOFs can improve charge transfer dynamics and create specific binding sites for target molecules.^19^ Upon light excitation, the organic linker of the MOF can act as a light absorber, and the MOF can undergo photo-induced charge dissociation as electrons fill the inorganic structural units and holes fill the organic linker. However, MOFs have a wide band gap, which limits their photocatalytic activity. Furthermore, significant charge recombination is observed in most MOFs. In combination with CQDs, MOFs act as both photosensitizers and electron acceptors, providing high efficiency in the degradation of organic pollutants.^20^ In this context, combining CQDs with MOF structures increases their load-carrying capacity and photocatalytic efficiency.^9,10,12,21–23^ Structures such as CQD@MIL125 (Ti)@ZIF67 have been shown to have great potential in areas such as pollutant treatment and photocatalytic degradation. However, properties such as anticancer and antibiofilm have also gained considerable importance in biological applications. Recently, CQDs and MOFs have been attracting significant attention in cancer treatment, in addition to their antifungal and antibacterial properties.^9,10,12^ MOF-on-MOF structures function as nanomolecules used in the diagnosis and treatment of cancer cells. The use of these materials in cancer treatment, based on both drug delivery capabilities and cancer cells themselves, has been shown to generate new solutions in both diagnosis and therapy.^24^ In conclusion, this study discusses the uses of CQD@MIL-125(Ti)@ZIF67 nanomaterials in various applications such as MOF-on-MOF structures, water treatment, cancer therapy, environmental protection, and biological sensing. Such nanomaterials are making significant progress in biomedical applications while enhancing environmental sustainability.

## 2. Materials and methods

### 2.1 Materials

Tetraisopropyl titanate (TIPT) (C_12_H_28_O_4_Ti), copper chloride (CuCl_2_), terephthalic acid (TA) (benzendicarboxylic acid, H_2_BDC; 98%), methanol (MeOH) (CH_3_OH; 99.9%), silver nitrate (AgNO_3_), iron(ii) chloride (FeCl_2_), iron(iii) chloride (FeCl_3_), zinc chloride (ZnCl_2_), calcium chloride (CaCl_2_), cadmium sulphide (CdS), manganese (ii) chloride (MnCl_2_), 2-methylimidazole (C_4_H_6_N_2_), nickel chloride (NiCl_2_), potassium chloride (KCl), sodium chloride (NaCl), aluminium chloride (AlCl_3_), cobalt nitrate hexahydrate (Co(NO_3_)_2_·6H_2_O), potassium dichromate (K_2_Cr_2_O_7_), all chemicals used in the experiments were sourced from Sigma Aldrich. Dimethylformamide (DMF) (99.9%) was sourced from ISOLAB. Bananas used in CQD synthesis were purchased from a local market.

### 2.2 Characterization

The characteristic properties of the CQD@ZIF-67@MIL-125(Ti) composite obtained by the green synthesis method were investigated using XRD, UV-VIS, FTIR, FE-SEM, and TEM techniques. The crystal lattice structure of the CQD@ZIF-67@MIL-125(Ti) composite was investigated using Rigaku MINI FLEX 600 (CuKα, *λ* = 1.5418 Å, 5–90°) and Panalytical EMPYREAN (CuKα, *λ* = 1.5405 Å, 5–90°) devices. The morphology and average particle size of the CQD@ZIF-67@MIL-125(Ti) composite structure were determined using a JEOL JEM 1220 100 kV TEM instrument and a Hitachi Regulus 8230 FE-SEM instrument, and the average particle sizes were calculated using the ImageJ program. To control the presence of organic chemicals in the composite material obtained by the green synthesis method, characterization was performed in the 400–4000 cm^−1^ range using a PerkinElmer FTIR Spectrophotometer. PerkinElmer Lambda 750 spectrophotometer was used for UV-VIS analysis. Fluorescence spectra of the synthesized composite were obtained using a PerkinElmer LS55 instrument.

### 2.3 Synthesis of CQD@ZIF-67@MIL-125(Ti)

1 mL of TIPT (3.38 mmol) was added to 5 mL of MeOH to obtain mixture A. 1 g of TA (5.5 mmol) was dissolved in 10 mL of DMF to obtain mixture B. Mixture A was slowly added to mixture B at room temperature, and the mixture was transferred to a 100 mL Teflon-coated stainless steel autoclave. It was incubated at 150 °C for 20 hours, and the resulting solid sample was centrifuged and washed several times with DMF and MeOH. Finally, the sample obtained for activation was dried in a vacuum oven at 80 °C for 24 hours.^21^ For the ZIF-MIL composite, 733 mg Co (NO_3_)_2_·6H_2_O was dissolved in 50 mL MeOH, and 100 mg MIL-125(Ti) was added to the solution and left to stand for 1 hour under sonication. 1.623 g of 2-methylimidazole was added to 50 mL of MeOH and dissolved at room temperature. Then, 2-methylimidazole solution was rapidly added to the ZIF-MIL solution and stirred for 8 hours. After 8 hours, the samples were centrifuged, washed three times with MeOH, and dried in a vacuum oven at 60 °C for 12 hours.^21^ For CQD synthesis, banana peels were cleaned, washed, and sun-dried. Approximately 3 g of the dried banana peels were placed in a Teflon-coated stainless-steel autoclave, and 50 mL of DS was added. It was then incubated at 200 °C for 8 hours. The resulting brown liquid was filtered through a 0.22 µm filter and stored at +4 °C. For the synthesis of CQD@ZIF-67@MIL125(Ti), 5 mL of CQD and 50 mg of ZIF-67@MIL-125(Ti) composite were mixed at room temperature for 4 hours. After 4 hours, it was centrifuged and washed three times with DS. Then it was dried in a vacuum oven at 60 °C for 4 hours.

### 2.4 CQD@ZIF-67@MIL-125(Ti) sensor analysis

5 µL of the composite was added to 3000 µL of DS and used as a control. Then, 5 µL of different metal ions were added, and the changes in the FL spectrum were examined. The synthesized composite was found to detect Cr^6+^ metal ions, and different concentrations of Cr^6+^ metal ions were investigated. Furthermore, Cr^6+^ metal ions (71 µM) and other metal ions (71 µM) were tested together. A pH study was also conducted to determine the optimum conditions for the FL sensor. The sensor's detection of Cr^6+^ in different water samples was also investigated.

## 3. Result and discussion

Characterization of the synthesized CQD@ZIF-67@MIL-125(Ti) composite was performed using various analytical techniques including XRD, TEM, FE-SEM, FL spectroscopy, UV-VIS spectroscopy, and FTIR. Furthermore, the results regarding the fluorescent sensor properties of the CQD@ZIF-67@MIL-125(Ti) composite for Cr^6+^ metal ion detection, its photocatalytic activity for the removal of MB dye from wastewater, and its biological activity, including antibacterial and anticancer properties, are presented.

### 3.1 FTIR analysis

**Fig. 1:**
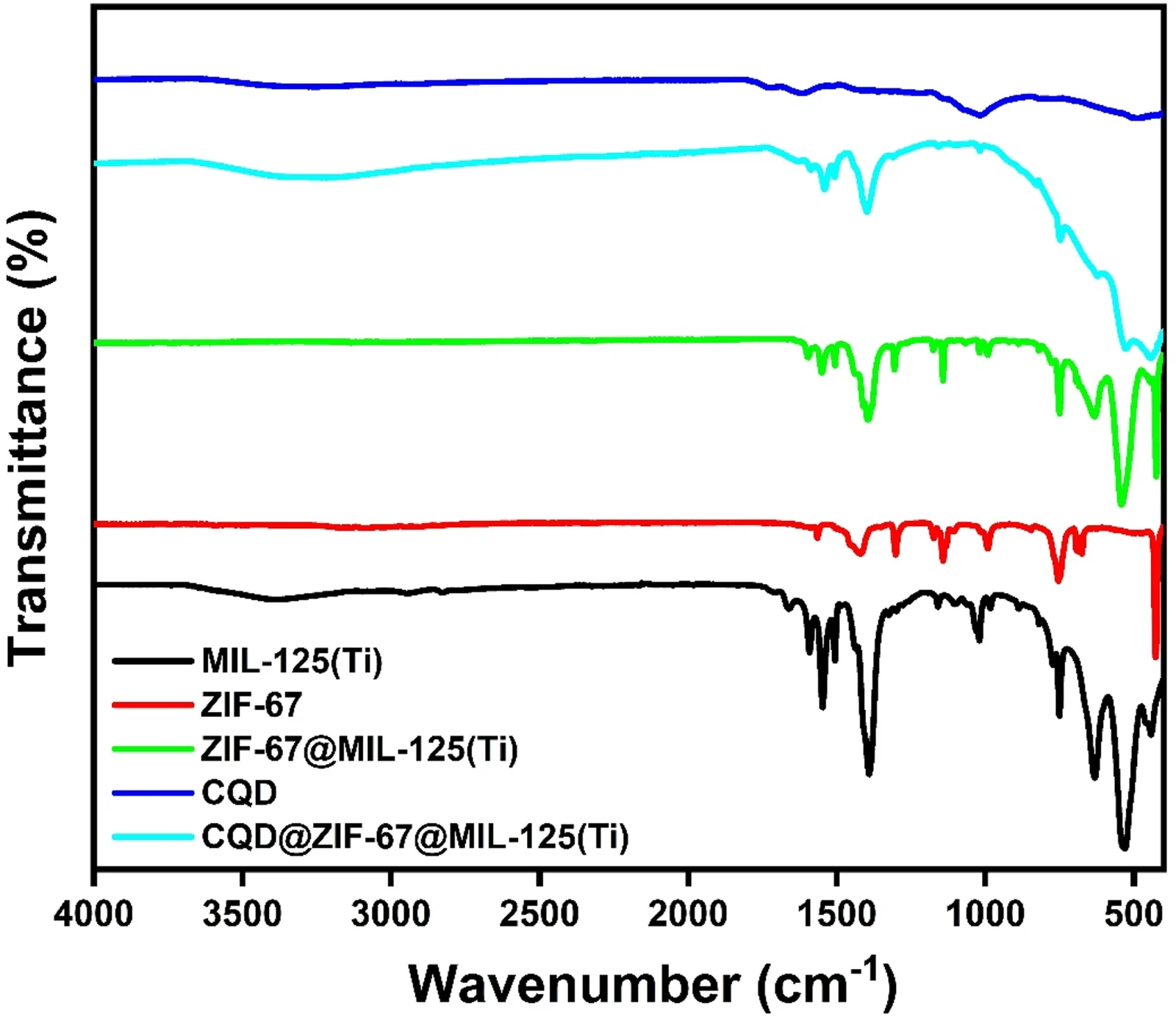
FTIR Spectra of MIL-125(Ti), ZIF-67, ZIF-67@MIL-125(Ti) and CQD@ZIF-67@MIL-125(Ti).

The peaks between 400–800 cm^−1^ in the FTIR spectrum of MIL-125(Ti) correspond to O–Ti–O vibrations. The peaks in the 1000–1300 cm^−1^ and 1500–1700 cm^−1^ ranges of MIL-125(Ti) can be attributed to C–O vibrations and –COOH asymmetric stretching vibration, respectively.^25^ In the FTIR spectrum of the ZIF-67 structure, the peak at 2922.8 cm^−1^ indicates the stretching vibration of the aliphatic C–H of methyl imidazole. The peaks at 1633 and 1416 cm^−1^ are associated with the C=N and C=C groups of imidazole, respectively. Additionally, the peak of 3439 cm^−1^ is associated with hydroxyl groups (O–H), and the peak of 425 cm^−1^ corresponds to the vibration of the Co–N bond. Peaks in the range of 600–1500 cm^−1^ correspond to the characteristic stretching and bending modes of the imidazole ring (Fig. 1). Furthermore, the peak at 1574 cm^−1^ corresponds to the C=N stress mode in 2-mim. The peaks at 2922 and 3130 cm^−1^ correspond to the stretching mode from the aromatic ring C–H and the aliphatic chain of 2-mim, respectively.^26^ Examination of the FTIR spectra of the prepared composites confirmed the presence of each structure they contained (Fig. 1).^9^

### 3.2 FE-SEM and TEM analysis

**Fig. 2:**
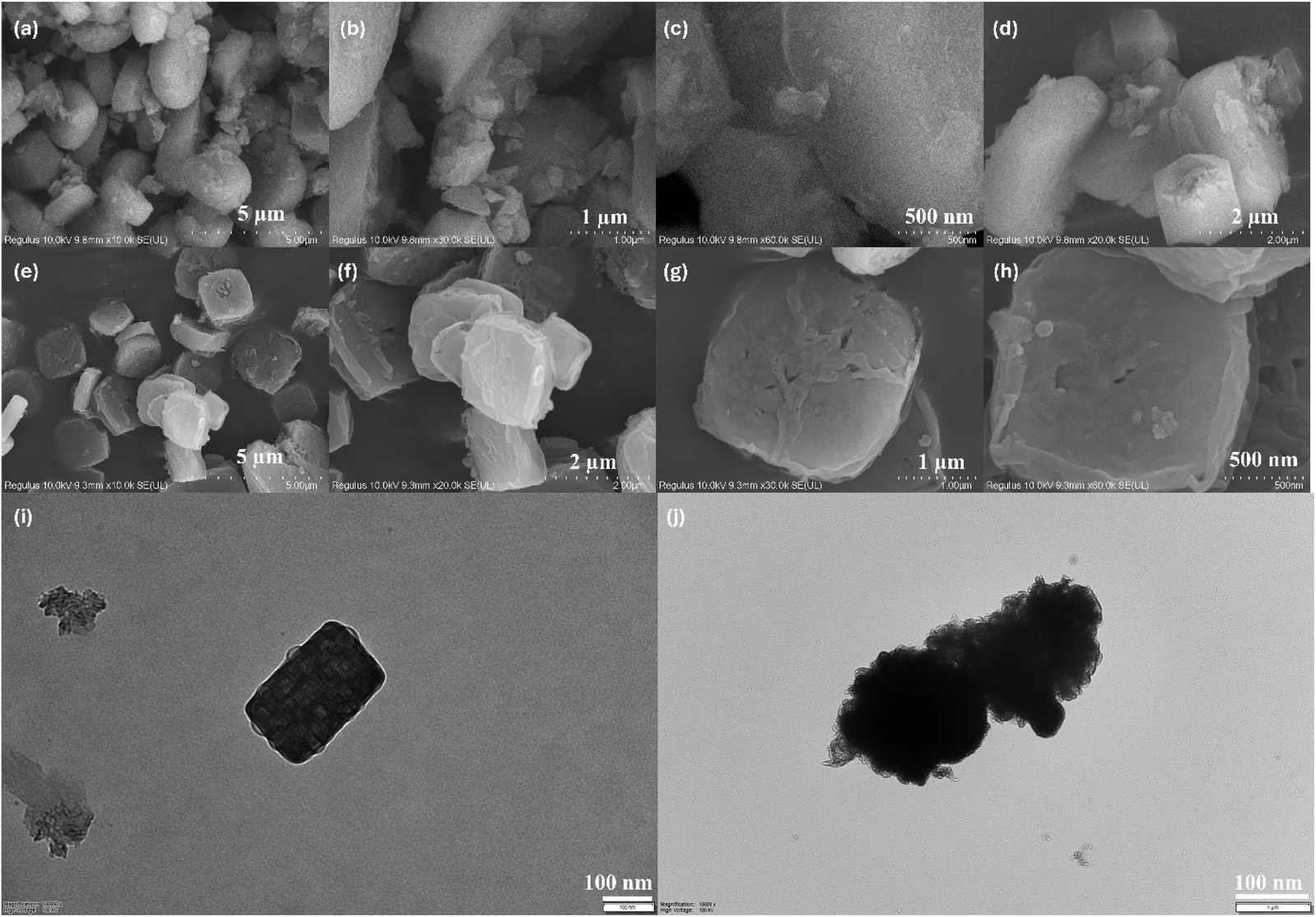
(a–d) FE-SEM Analysis of ZIF-67@MIL-125(Ti) and (e–h) CQD@ZIF-67@MIL-125(Ti) and TEM Analysis of (i) ZIF-67@MIL-125(Ti) and (j) CQD@ZIF-67@MIL-125(Ti).

**Fig. 3:**
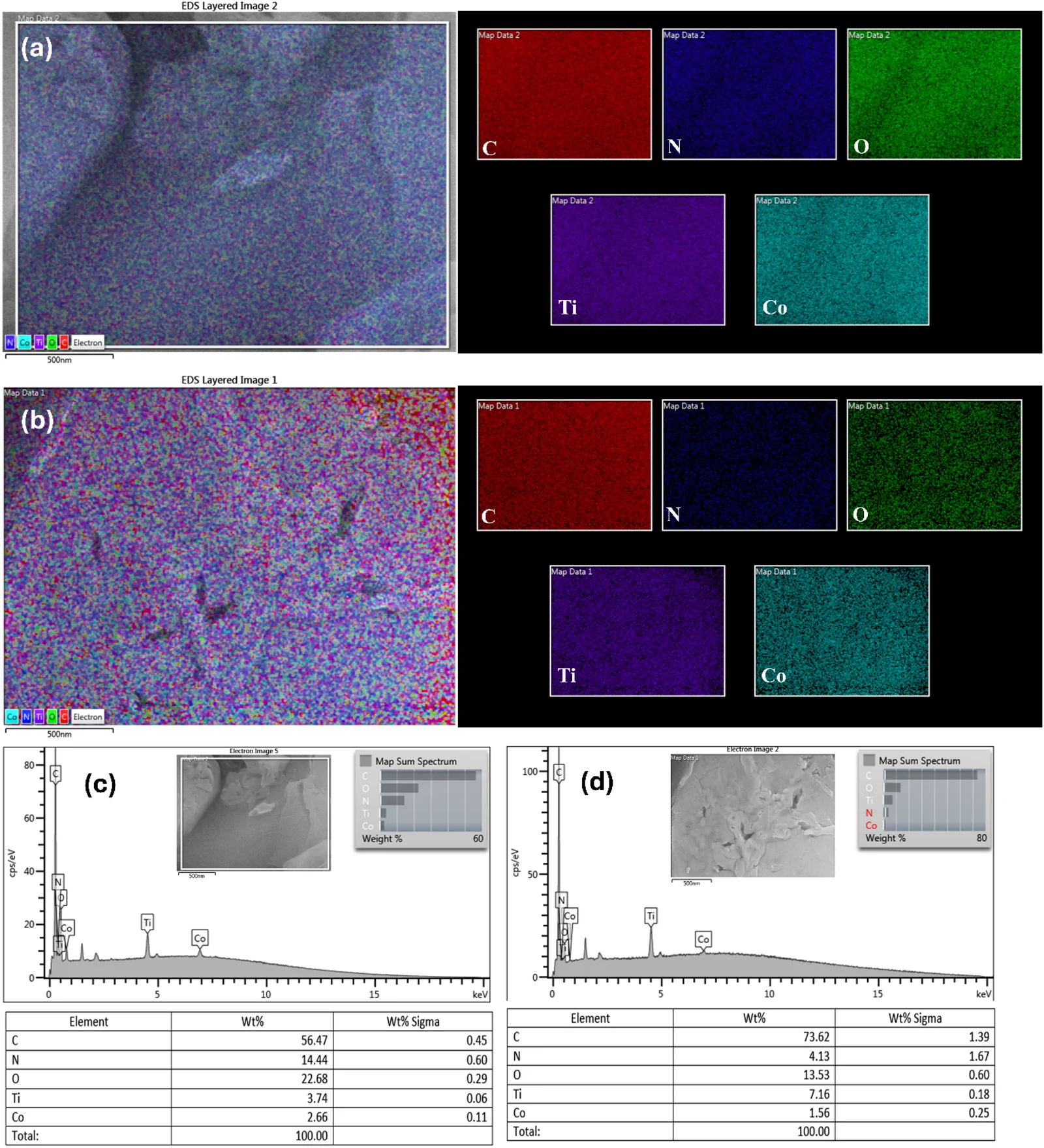
(a) EDX Mapping Images of ZIF-67@MIL-125(Ti) and (b) CQD@ZIF-67@MIL-125(Ti) and (c) EDX Spectrum of ZIF-67@MIL-125(Ti) and (d) CQD@ZIF-67@MIL-125(Ti).

Fig. 2 shows FE-SEM and TEM analysis images illustrating the unique and characteristic morphology of CQD@ZIF-67@MIL-125(Ti). ZIF-67 nanocrystals were grown directly onto MIL-125(Ti) without the use of surfactants, and MOF-on-MOF heterostructures were formed. Fig. 2(a–d) shows the FE-SEM images of ZIF-67@MIL-125(Ti), and it is observed that the ZIF-67 structures are concentrated on the upper surface of MIL-125(Ti). The particle shape has been identified as typical circular plates. The EDX analysis in Fig. 3(a) shows the presence of both Ti and Co, meaning that ZIF-67 is well incorporated into MIL-125(Ti).^9,21^ With subsequent CQD loading, the structure became more angular and layered, indicating that CQDs strongly interact with the surface of the MIL-125(Ti) structure, driving crystal growth (Fig. 2(e–h)). The EDX analysis in Fig. 3(b) shows the carbon density on the surface of the composite structure and CQDs are incorporated into the ZIF-67@MIL-125(Ti) MOF-on-MOF structure. However, in the CQD@ZIF-67@MIL-125(Ti) EDX spectrum, a decrease in Co percentage was observed (Fig. 3(c)), while an increase in C percentage occurred (Fig. 3(d)), and this is due to the fact that CQDs alter the surface morphology, differentiating the relative ratios of the elements. TEM analysis of CQDs is shown in Figure S1. TEM and FE-SEM images confirm the composite structure. The incorporation of CQDs into the MOF-on-MOF structure did not cause any changes in the crystal structure.

### 3.3 XRD analysis

**Fig. 4:**
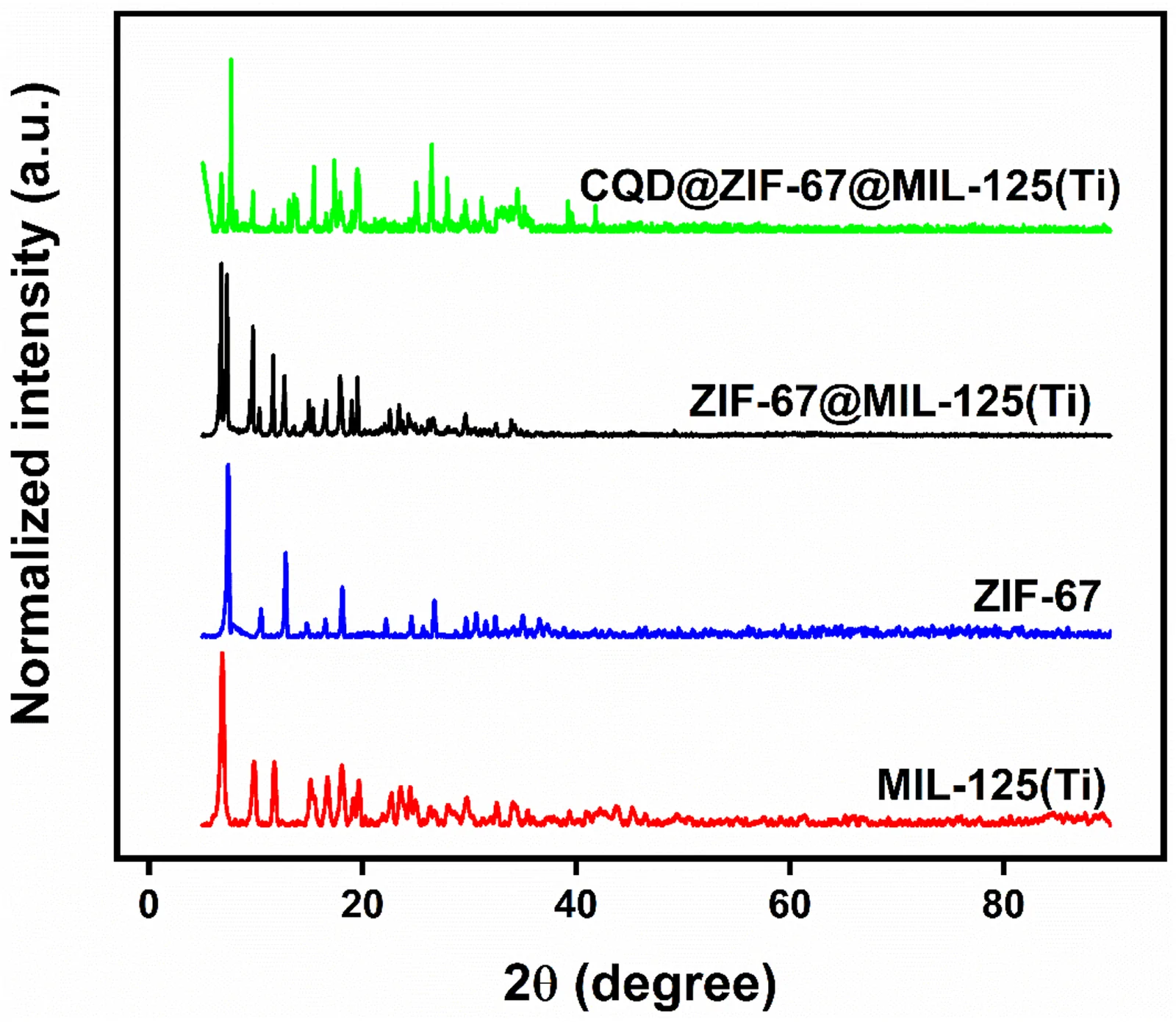
XRD Analysis of MIL-125(Ti), ZIF-67, ZIF-67@MIL-125(Ti) and CQD@ZIF-67@MIL-125(Ti).

The XRD spectra of CQD@ZIF-67@MIL-125(Ti), ZIF-67@MIL-125(Ti), MIL-125(Ti), and ZIF-67 are shown in Fig. 4. The peaks observed at 6.7°, 9.7°, 11.6°, 15.2°, 16.6°, 17.9°, 19.5°, 21.5°, 22.6° and 25.3° in the XRD spectrum of MIL-125(Ti) are consistent with previously reported MIL-125(Ti).^27,28^ ZIF-67 shows peaks at 7.42° (011), 10.52° (002), 12.81° (112), 14.77° (002), 16.50° (013), 18.17° (222), 22.15° (114), 24.54° (233), 26.68° (134) and 29.77° (004), 30.63° (334), 31.5° (244), 32.34° (235), 43.19° (100) and is associated with the previously reported ZIF-67.^29^ In the XRD spectrum of ZIF-67@MIL-125(Ti), it was determined that peaks belonging to both MIL-125(Ti) and ZIF-67 structures were present together.^9^ It was determined that there was no significant loss of crystallinity after adding ZIF-67 to the MIL-125(Ti) matrix.^21^ The XRD spectrum of CQD@MIL-125@ZIF-67 in the 5–90° 2θ range is like the spectrum of ZIF-67@MIL-125(Ti). These results show that the MOF-on-MOF crystal structure remains unchanged due to the inclusion of CQDs.^5^

### 3.4 FL Spektrum analysis

**Fig. 5:**
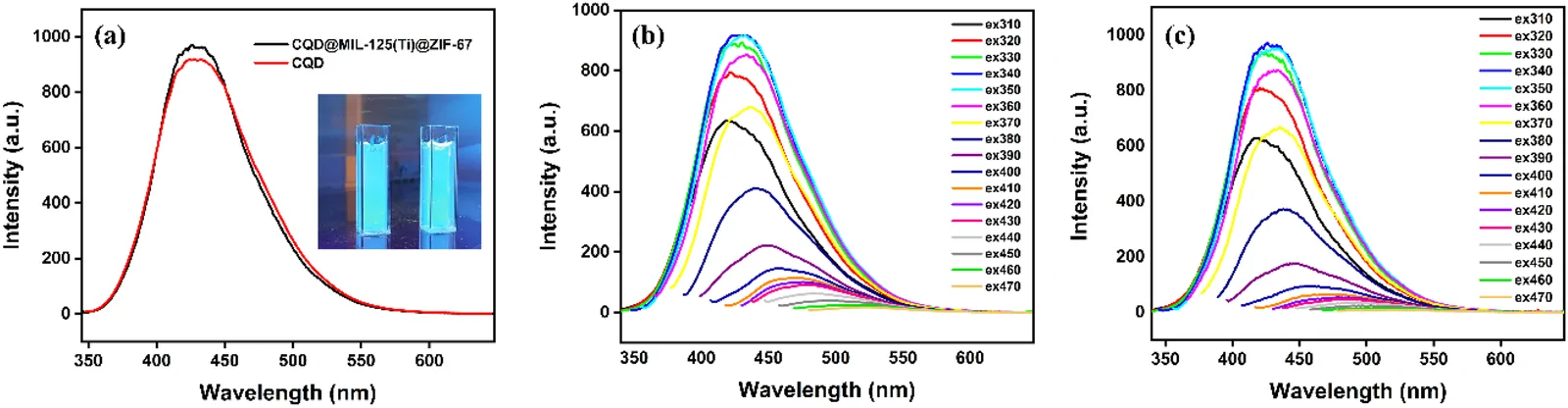
(a) FL spectra of CQD and CQD@ZIF-67@MIL-125(Ti) (b) FL Spectra of CQD and (c) FL Spectra of CQD@ZIF-67@MIL-125(Ti) at different excitation wavelengths (310–470 nm, in 10 nm intervals)

The FL spectrum in Fig. 5(a) shows that the FL emission wavelength of the CQDs reaches a maximum emission at 428 nm with an excitation wavelength of 440 nm. The FL spectra of CQD@ZIF-67@MIL-125(Ti) show similarities to those of CQD, and doping of CQD onto the ZIF-67@MIL-125(Ti) surface resulted in a slight increase in FL intensity. Fig. 5(b) and (c) show the FL spectra of CQD and CQD@ZIF-67@MIL-125(Ti) at different excitation wavelengths. The FL phenomenon, a fundamental characteristic of CQDs, is a consequence of quantum effects.^30^ A gradual bathochromic (red) shift in emission was observed in the blue region, depending on the excitation wavelength of the strong emission peaks. One known characteristic of CQDs is excitation-induced emission variation. The existence of different particle sizes of CQDs can explain the fluorescence behavior of CQDs. The observation of differences in particle sizes in CQDs occurs through quantum confinement effects and π-electron delocalization, which can either increase or decrease the energy band gap. The FL mechanism of CQDs depends on many factors and is quite complex. These factors include the presence of various functional groups and surface defects on the surface. Generally, we can say that the particle size and surface defects of CQDs directly affect the fluorescence mechanism. Furthermore, doping CQDs with different atoms results in significant changes in fluorescence emission.^31^

### 3.5 Photocatalytic activity results

**Fig. 6:**
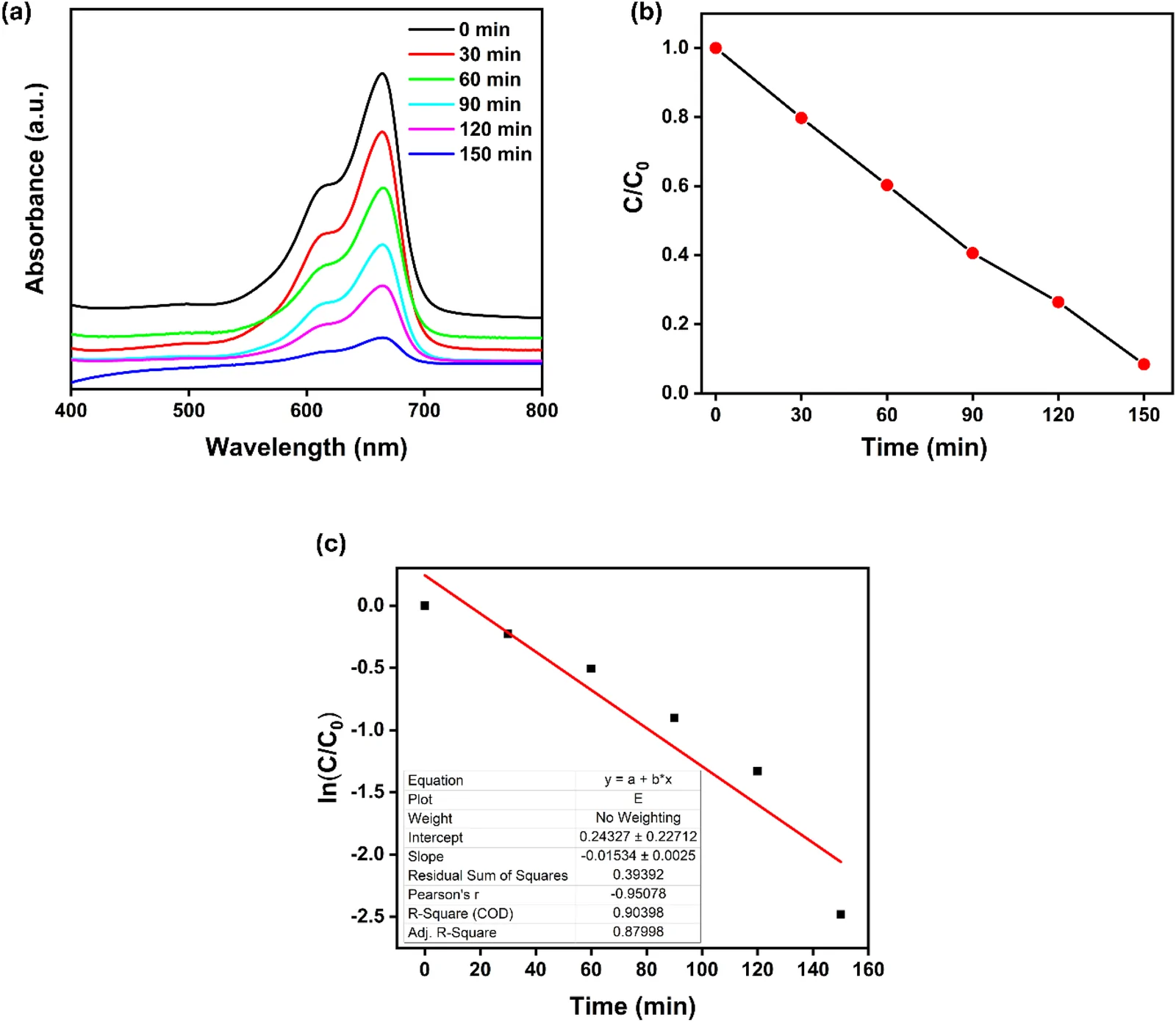
(a) Time-dependent absorbance spectra of MB (b) time-dependent *C*/*C*_0_ ratio of MB (c) kinetic graph of MB degradation.

The photocatalytic activity of the synthesized CQD@ZIF-67@MIL-125(Ti) composite was tested against MB dye under visible light. Fig. 6(a) shows the UV-VIS spectrum of MB, and it can be said that MB degrades in the presence of a photocatalyst under visible light. Fig. 6(a) shows that the characteristic absorbance peak of MB decreases over time. After 150 minutes, it was determined that MB had degraded by 91.63%. Fig. 6(b) shows the photocatalytic activity of CQD@ZIF-67@MIL-125(Ti) towards the photodegradation of MB. It is estimated that the photocatalytic degradation kinetics of MB in the presence of CQD@ZIF-67@MIL-125(Ti) are pseudo-first-order. In Fig. 6(c), the kinetics of the degradation reaction were calculated using eqn (1). The photocatalytic decomposition reaction of MB supports pseudo-first-order kinetics, as the linear graph of ln(*C*/*C*_0_) *versus* reaction time (*R*_2_ = 0.9039). The degradation reaction rate constant, indicating the photocatalytic activity of the synthesized CQD@ZIF-67@MIL-125(Ti) composite, was found to be *k* = 0.015 min^−1^.

1 −ln(*C*/*C*_0_) = *kt*

**Fig. 7:**
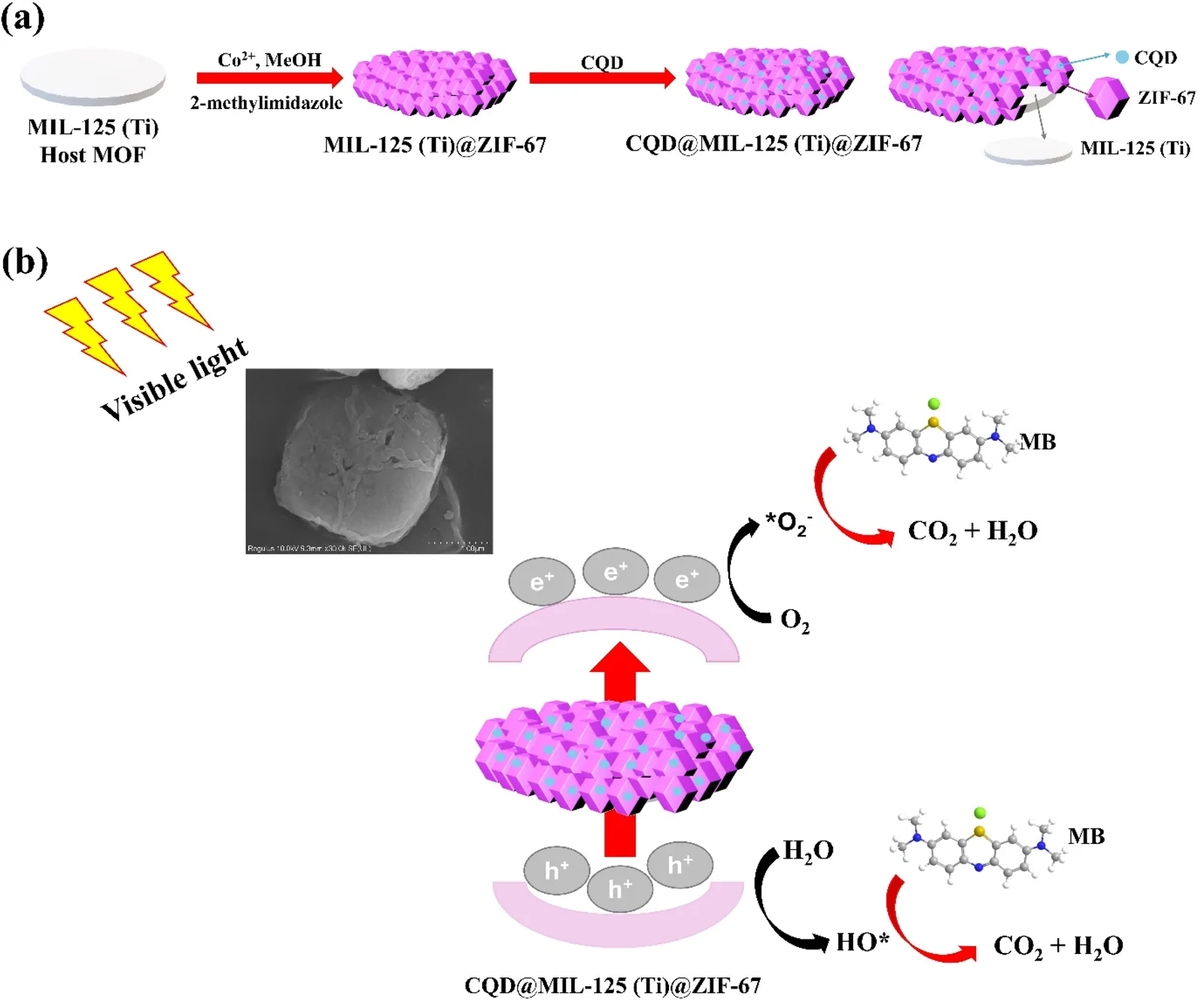
(a) Illustration of the synthesis process of CQD@ZIF-67@MIL-125(Ti) (b) possible photodegradation mechanism of MB in the presence of CQD@ZIF-67@MIL-125(Ti) under visible light.

Fig. 7(a) shows the synthesis step of the MOF-on-MOF heterostructure. MIL-125(Ti), which has a cake-like morphology, was chosen as the host for the MOF-on-MOF structure. Then, ZIF-67 was *in situ* grown on this host. After obtaining the MOF-on-MOF structure, CQD was added to obtain CQD@ZIF-67@MIL-125(Ti). Fig. 7(b) shows the possible mechanism for the photodegradation of MB. Electron–hole separation occurred in all three components of CQD@ZIF-67@MIL-125(Ti). The surface of the catalyst, characterized by high electron density, interacts with oxygen molecules (O_2_) to form superoxide radicals (^.^O_2_^−^), while the hole structures interact with water molecules or remove electrons from hydroxyl ions to form hydroxyl radicals (HO˙). The radicals ˙O_2_^−^ and HO⋅ are involved in the decomposition of MB and the formation of CO_2_ and H_2_O as end products. The presence of CQD enhances charge transfer between ZIF-67@MIL-125(Ti) and can effectively inhibit e^−^/h^+^ recombination in the reaction system.

### 3.6 Antibacterial activity

**Fig. 8:**
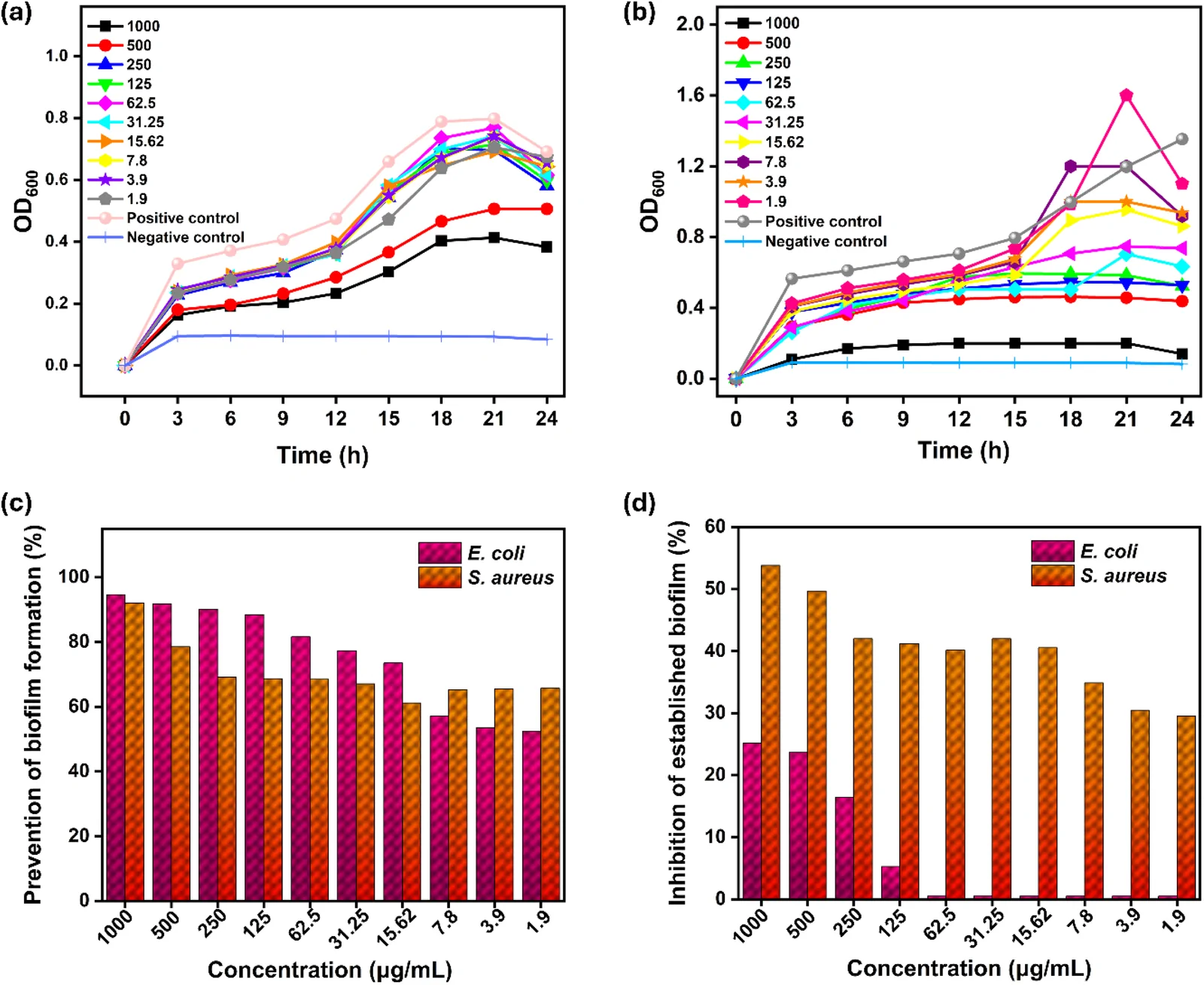
(a) OD values at 600 nm wavelength for *E. coli* bacteria and (b) OD values at 600 nm wavelength for *S. aureus* bacteria (c) prevention of biofilm formation by *E. coli* and *S. aureus* bacteria (%) (d) inhibition of established biofilm by *E. coli* and *S. aureus* bacteria (%).

The antibacterial and antibiofilm activity of the synthesized CQD@ZIF-67@MIL-125(Ti) was evaluated against *E. coli* and *S. aureus* bacteria. Fig. 8(a) shows the growth curves of *E. coli* treated with CQD@ZIF-67@MIL-125(Ti) as a function of time and sample concentration. The negative control contained only the culture medium and had a very low OD_600_ value. The positive control contained bacterial culture without a sample, and the OD_600_ value was observed to be higher than all concentrations. Fig. 8(b) shows the time and sample concentration dependent growth curves of *E. coli* treated with CQD@ZIF-67@MIL-125(Ti). The negative control contains only the culture medium and has a very low OD_600_ value. The positive control contains the bacterial culture without the sample and the OD_600_ value was observed to be above all concentrations. Fig. 8(b) shows the time and sample concentration dependent growth curves of *S. aureus* treated with CQD@ZIF-67@MIL-125(Ti). Fig. 8(a) and (b) show that CQD@ZIF-67@MIL-125(Ti) limits and inhibits the growth of both *E. coli* and *S. aureus* bacteria. MIC values for *E. coli* and *S. aureus* were found to be 500 µg mL^−1^ and 125 µg mL^−1^, respectively. Fig. 8(c) shows the potential of CQD@ZIF-67@MIL-125(Ti) to inhibit biofilm formation. The biofilm formation inhibition rate of CQD@ZIF-67@MIL-125(Ti) for *E. coli* and *S. aureus* was calculated as 94.62% and 92.05%, respectively. Fig. 8(d) shows the biofilm inhibition potential of CQD@ZIF-67@MIL-125(Ti). The biofilm inhibition of CQD@ZIF-67@MIL-125(Ti) for *E. coli* and *S. aureus* was calculated as 25.13% and 53.78%, respectively. The results obtained show that CQD@ZIF-67@MIL-125(Ti) has independent antibacterial and antibiofilm activities. In inhibiting bacteria grown in liquid medium, a greater effect was observed for *S. aureus*. CQD@ZIF-67@MIL-125(Ti) was highly effective in inhibiting biofilm formation for both bacteria, while showing efficacy in bacterial inhibition for *S. aureus*. For *E. coli*, only 25.13% inhibition was observed at a concentration of 1000 µg mL^−1^, and no effect was observed at concentrations lower than 125 µg mL^−1^. All three components are thought to be effective in the antibacterial activity mechanism. The release of Co^2+^ and Ti^4+^ metal ions from the binary MOF composite and the release of different organic linkers (2-methylimidazole and terephthalic acid) present in the composites may cause damage to the bacterial cell membrane and cell wall, as well as damage to bacterial enzyme systems and DNA.^9^ Due to their small size and functional groups, CQDs can make bacterial cell membranes permeable. CQDs can promote the production of ROS species through electrostatic interaction with bacterial cells.^32,33^ Synthesis of catalysts containing the ZIF-67@ZIF-8@MIL-125-NH_2_ ternary MOF component has been reported in the literature. The MIC values of ZIF-67@ZIF-8@MIL-125-NH_2_ against *S. aureus* ATCC 6538, *Bacillus subtilis* ATTC 6051, *E. coli* ATCC 8739, *P. aeruginosa* ATCC 9027 and *S. typhimurium* ATCC 14028C bacteria were found to be 0.21 ± 0.10, 0.20 ± 0.01, 0.25 ± 0.03, 0.23 ± 0.02 and 0.24 ± 0.04 mg mL^−1^, respectively. The inhibition rates of *S. aureus* bacterial biofilm formation by ZIF-67@ZIF-8@MIL-125-NH_2_ at concentrations of 0.6, 0.3, 0.15, and 0.07 mg mL^−1^ were reported as 93.2%, 91.5%, 89.8%, and 86%, respectively. It has been reported that at concentrations below the MIC value, biofilm formation of *S. aureus* is inhibited while bacterial growth is not affected.^9^ The MIC values of the Thio@MIL-125-NH_2_@CMC composite against *S. aureus* and *E. coli* bacteria were found to be 250 and 1000 µg mL^−1^, respectively.^34^

### 3.7 Anticancer activity

**Fig. 9:**
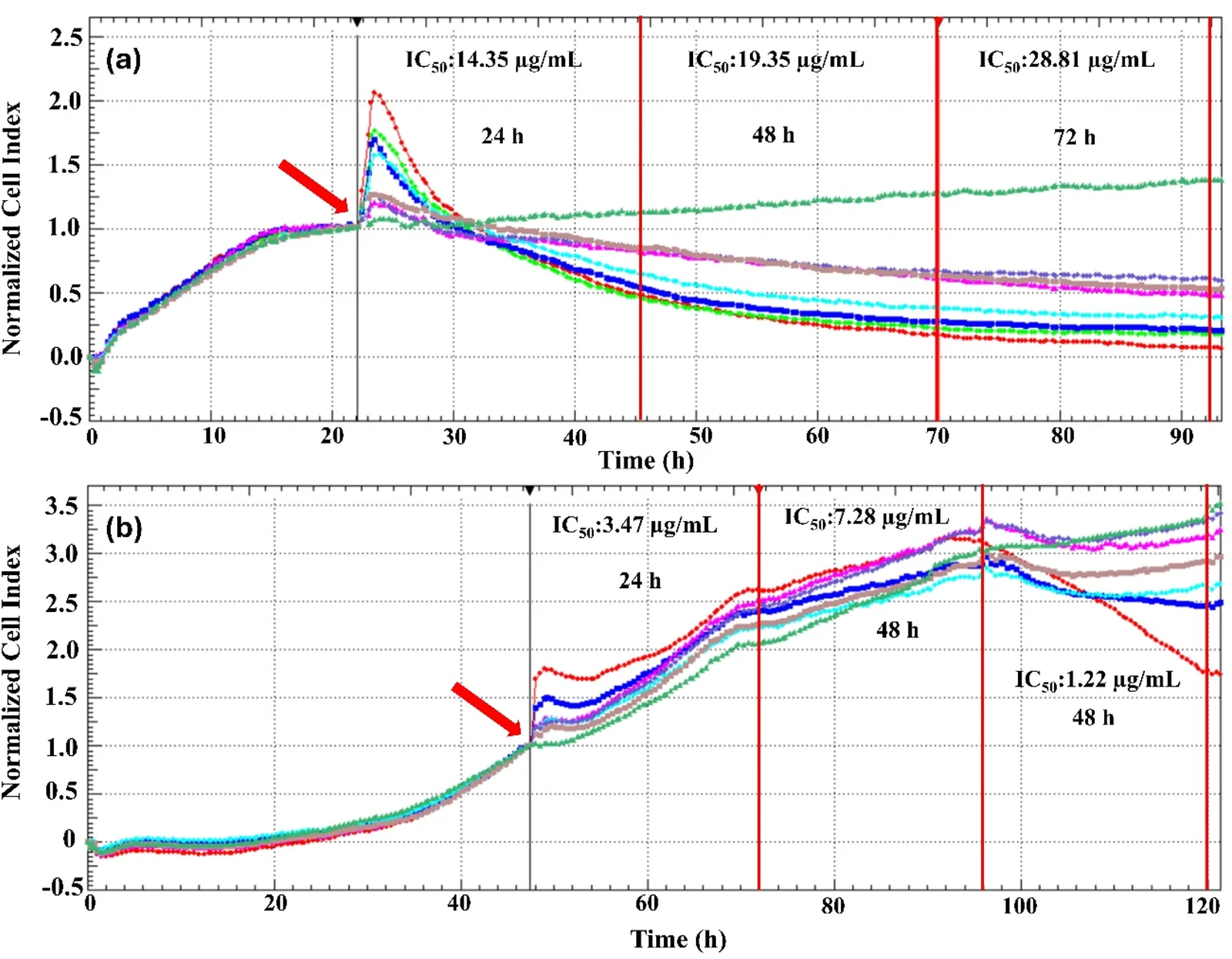
Growth curves of (a) HepG2 and (b) HT-29 cancer cells exposed to CQD@ZIF-67@MIL-125(Ti) concentrations between 2.5-250 µg mL^−1^ for 72 hours (line indicates the addition of composites and normalization time point).

The anticancer activity of CQD@ZIF-67@MIL-125(Ti) was investigated against HepG2 and HT-29 cancer cell lines. Time and concentration-dependent growth curves of HepG2 and HT-29 cancer cells treated with different concentrations of CQD@ZIF-67@MIL-125(Ti) are shown in Fig. 9(a) and (b). For the HepG2 cancer line, IC_50_ values at 24, 48, and 72 hours were found to be 14.35, 19.35, and 28.81 µg mL^−1^, respectively. The IC_50_ values for the HT-29 cancer line at 24, 48, and 72 hours were found to be 3.47, 7.28, and 1.22 µg mL^−1^, respectively. The results show that CQD@ZIF-67@MIL-125(Ti) is effective against both cancer types but was more effective against the HT-29 cancer cell line. In the literature, the IC_50_ value of biogenic CQDs obtained from *Typha angustifolia* by the hydrothermal method against the human breast cancer (MDA-MB-231) cell line has been reported as 70.55 ± 0.015 µg mL^−1^.^35^ The IC_50_ values for CCM (Curcumin), CCM-ZIF-67, and CCM-ZIF-8 against the HeLa cancer cell line were found to be 83.89, 30.55, and 47.75 µg mL^−1^, respectively.^36^ The IC_50_ value of cobalt oxide nanoparticles (Co_3_O_4_NPs) obtained by green synthesis using an aqueous extract of red algae against the HepG2 cancer cell line has been reported as 41.44 µg mL^−1^.^37^ IC_50_ values ​​of 29.73 and 34.27 µg mL^−1^ were obtained for TiO_2_ NPs in HeLa and HEK 293 cell lines using aqueous leaf extract of *Tulbaghia violacea*.^38^ The IC_50_ values ​​of α-Asarone-doped NH_2_-MIL-125 for A549 and WI-38 cell lines were found to be 5.65 ± 2.81 and 291.36 ± 1.95 µg mL^−1^, respectively.^39^ Ag@MOF has been reported to have IC_50_ values of 49 and 56 µg mL^−1^ against HCT-116 and HT-29 cancer cell lines, respectively.^40^

Increased intracellular reactive oxygen species (ROS) trigger a cascade that disrupts homeostatic balance by threatening the structural integrity of critical cellular components. The severity of dysfunction observed at the macromolecular level is directly related to the concentration of lipid peroxidation products and thiol balance parameters reflecting cellular redox potential. In this study, oxidative stress biomarkers, including total GSH levels, catalase activity, ROS production, and malondialdehyde levels (a marker of cell membrane lipid peroxidation), were evaluated in HepG2 and HT-29 cells following exposure to CQD@ZIF-67@MIL-125(Ti). Catalase enzyme activity was increased in both cell types compared to the control (*p* < 0.05). Total GSH and MDA levels, which indicate lipid peroxidation, were decreased in both cell types compared to the control (*p* < 0.05). The results obtained are shown in Fig. S2.

### 3.8 Fluorescent sensor characteristics for Cr^6+^ detection of CQD@ZIF-67@MIL-125(Ti)s

**Fig. 10:**
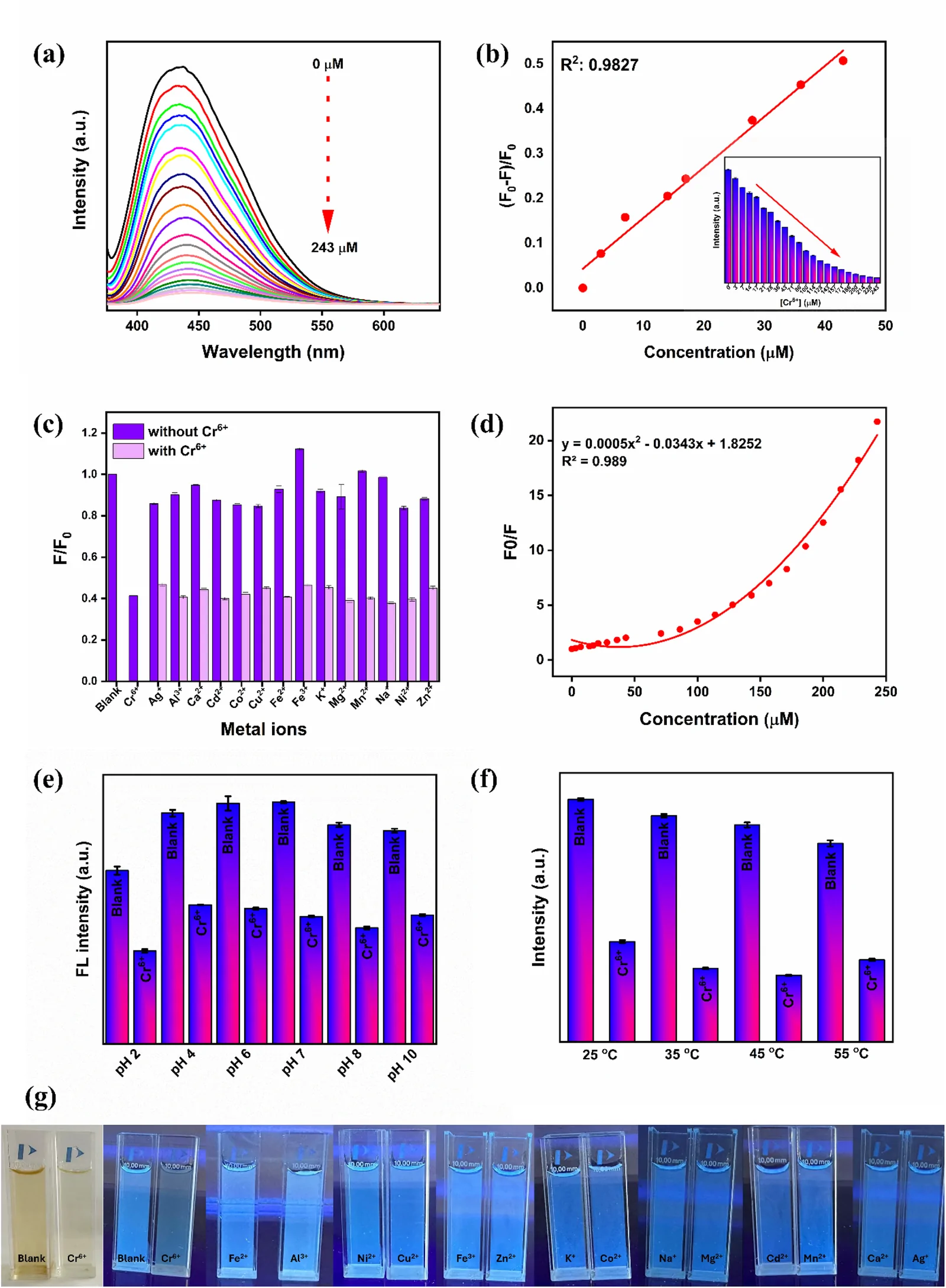
(a) Fluorescence spectra of CQD@ZIF-67@MIL-125(Ti) with different Cr^6+^ concentrations (0–243 µM) (b) calibration curve of Cr^6+^ concentration against fluorescence quenching of CQD@ZIF-67@MIL-125(Ti) (c) selectivity and interference of CQD@ZIF-67@MIL-125(Ti) towards Cr^6+^ (d) Stern–Volmer quenching graph derived from FL spectra of CQD@ZIF-67@MIL-125(Ti) against different concentrations of Cr^6+^ (e) effect of pH on fluorescence intensity of CQD@ZIF-67@MIL-125(Ti) (f) effect of temperature effect of CQD@ZIF-67@MIL-125(Ti)s on fluorescence intensity (g) fluorescence response of CQD@ZIF-67@MIL-125(Ti)s containing different metal ions under UV light.

To determine the sensor activity of CQD@ZIF-67@MIL-125(Ti)s for the detection of Cr^6+^, they were treated with different concentrations of Cr^6+^ and FL spectra were recorded. Fig. 10(a) shows that increasing Cr^6+^ concentrations from 3 µM to 243 µM causes a significant decrease in the emission intensity of CQD@ZIF-67@MIL-125(Ti). As the Cr^6+^ concentration decreased, a shift towards the red region occurred in the emission peak. This may be due to the response of blue-emitting CQDs to Cr^6+^. Responses of the (*F*_0_ − *F*)/*F*_0_ fluorescence intensity ratio were obtained with various Cr^6+^ concentrations. Fig. 10(b) shows that a linear relationship in the Cr^6+^ concentration range of 3 to 43 µM, with the calibration curve equation expressed as *Y* = 0.0427*X* + 0.011 and correlation coefficient *R*^2^ of 0.9827. Additionally, Fig. 10(b) shows the histogram graph indicating that FL intensity decreases depending on Cr^6+^ concentration. The LOD value for Cr^6+^ detection of CQD@ZIF-67@MIL-125(Ti) was calculated as 3.28 µM. Fig. 10(c) shows that when various metal ions, including Na^+^, K^+^, Ca^2+^, Mg^2+^, Al^3+^, Mn^2+^, Co^2+^, Ni^2+^, Cu^2+^, Zn^2+^, Cd^2+^, Fe^3+^, Fe^2+^ and Ag^+^, are added to CQD@ZIF-67@MIL-125(Ti), there is no significant change in fluorescence emission. Furthermore, it was determined that the addition of different metal ions in the presence of Cr^6+^ interfered very little with the recognition of Cr^6+^ (Fig. 10(c)). The results showed that all tested metal ions had a negligible effect on the fluorescence response of CQD@ZIF-67@MIL-125(Ti)s to Cr^6+^. Fig. 10(c) showed that CQD@ZIF-67@MIL-125(Ti) has a high selectivity for Cr^6+^ against other competing metal ions. Generally, quenching mechanisms can be divided into two main categories: dynamic and static. Static quenching involves the formation of a radiation-free complex between the fluorophore and the analyte while they are in their ground states. In this mechanism, the intrinsic lifetime of the sensor system is not affected even if the analyte concentration increases. Dynamic quenching is characterized by electron transfer due to collisions between the excited fluorophore and the quenching molecule. This collision phenomenon reduces the average lifetime of the fluorophore as the quenching concentration increases.^41^Fig. 10(d) shows the Stern–Volmer plot. The sensitivity of fluorescent quenching can be determined by the Stern–Volmer plot.^42^ The sensitivity of fluorescent quenching is determined by plotting the Stern–Volmer graph given by eqn (2).

2 *F*_0_/*F* = 1 + *K*_q_*τ*_0_[Q] = 1 + *K*_SV_[Q]

In this formulation, *F*_0_ and *F* correspond to the fluorescence intensity of CQD@ZIF-67@MIL-125(Ti) at approximately 437 nm in the absence and presence of Cr^6+^, respectively. [Q] is the Cr^6+^ concentration, and KSV is the Stern–Volmer quenching constant, which is related to the quenching efficiency.^43^ As shown in Fig. 10(d), a parabolic relationship with a correlation coefficient of *R*^2^ = 0.989 was observed between the fluorescence intensity ratios (*F*_0_/*F*) and Cr^6+^ concentration in the range of 0 to 243 µM. In Fig. 10(d), it was observed that the increase in the *F*_0_/*F* value was limited at low Cr^6+^ concentrations, while the *F*_0_/*F* value increased significantly at high Cr^6+^ concentrations. This may be related to the concentration-dependent increase in interactions between the probe and the high Cr^6+^ concentration, and the increasing contribution of different quenching processes. This result suggests that different quenching mechanisms may occur at low and high Cr^6+^ concentrations. The decrease in fluorescence intensity is thought to be due to the quenching of fluorescence centers as a result of interactions between functional groups on the surface of CQDs and Cr^6+^. Furthermore, it is thought that MOF structures, due to their high porosity and large surface area, facilitate the access and interaction of Cr ions with CQDs. The upward deviation from linearity in the Stern–Volmer quenching plot shown in Fig. 10(d) suggests that static and dynamic quenching may contribute together.^44^ The stability and fluorescence properties of CQD@ZIF-67@MIL-125(Ti) sensors were investigated under pH conditions ranging from 2 to 10. pH variations can significantly affect the sensitivity and selectivity of the sensor to metal ions in biological or environmental matrices. Fig. 10(e) shows the effect of pH on FL intensity and fluorescence quenching of CQD@ZIF-67@MIL-125(Ti) in the presence of 71 µM Cr^6+^. It was determined that the pH dependence of FL also exhibited the same trend in the absence of Cr^6+^. However, the fluorescence intensity of CQD@ZIF-67@MIL-125(Ti) showed minimal change over a wide pH range, with the highest fluorescence intensity observed at pH 7. Fig. 10(f) shows the effect of temperature on the fluorescence intensity and Cr^6+^ detection of CQD@ZIF-67@MIL-125(Ti) at different temperature values. Although the fluorescence intensity does not seem to fluctuate much, the results show that room temperature is the best operating temperature for CQD@ZIF-67@MIL-125(Ti). Furthermore, CQD@ZIF-67@MIL-125(Ti)s were successful in detecting Cr^6+^ at all studied temperature values. Fig. 10(g) shows the fluorescence responses of CQD@ZIF-67@MIL-125(Ti)s under UV light in the presence of different metal ions. It was observed that CQD@ZIF-67@MIL-125(Ti)s glowed under UV light and continued to glow in the presence of metal ions other than Cr^6+^. The fluorescence of CQD@ZIF-67@MIL-125(Ti)s was suppressed because of interaction with Cr^6+^. Table 1 shows a comparison of the LOD values of the CQD@ZIF-67@MIL-125(Ti) fluorescence probe with other sensor probes in the literature. Considering the LOD value, it can be emphasized that the synthesized sensor probe is designed for industrial wastewater (textile, metal plating, leather processing, *etc.*) containing much higher concentrations of Cr, rather than for detecting Cr concentrations in ultra-pure drinking water.

**Table 1:** Comparison of Cr^6+^ detection performance of CQD@ZIF-67@MIL-125(Ti) with other sensor materials in the literature

Sensing material	Detection method	Analyte	LOD	References
N-doped Co_3_O_4_/ZnO/NiO S-scheme	Fluorescence	Cr^6+^	5.01 µM	45
N-doped CQDs	Fluorescence	Cr^6+^	19.37 µM	16
Cyanobacteria-derived CQDs	Fluorescence	Cr^6+^	0.6831 mg L^−1^	46
Zn-MOF {[Zn_2_(L1)(5-ATZ)]·1.5DMF·H_2_O}_n_	Fluorescence	Cr_2_O_7_^2-^	4 × 10^−4^ M	47
		CrO_4_^2-^	6.3 × 10^−4^ M	
MOF-derived 1D CeO_2_ nanobars	Fluorescence	Cr^6+^	0.58 µg mL^−1^	48
CQDs	Fluorescence	Cr^6+^	14 µM	49
Fe-QueNPs@ZIF-67	Colorimetric (nanozyme-based)	Cr^6+^	0.36 µM	50
ZIF-8	Fluorescence	Cr^6+^	4.7 × 10^−5^ M	51
CQD@ZIF-67@MIL-125(Ti)	Fluorescence	Cr^6+^	3.28 µM	This study

**Fig. 11:**
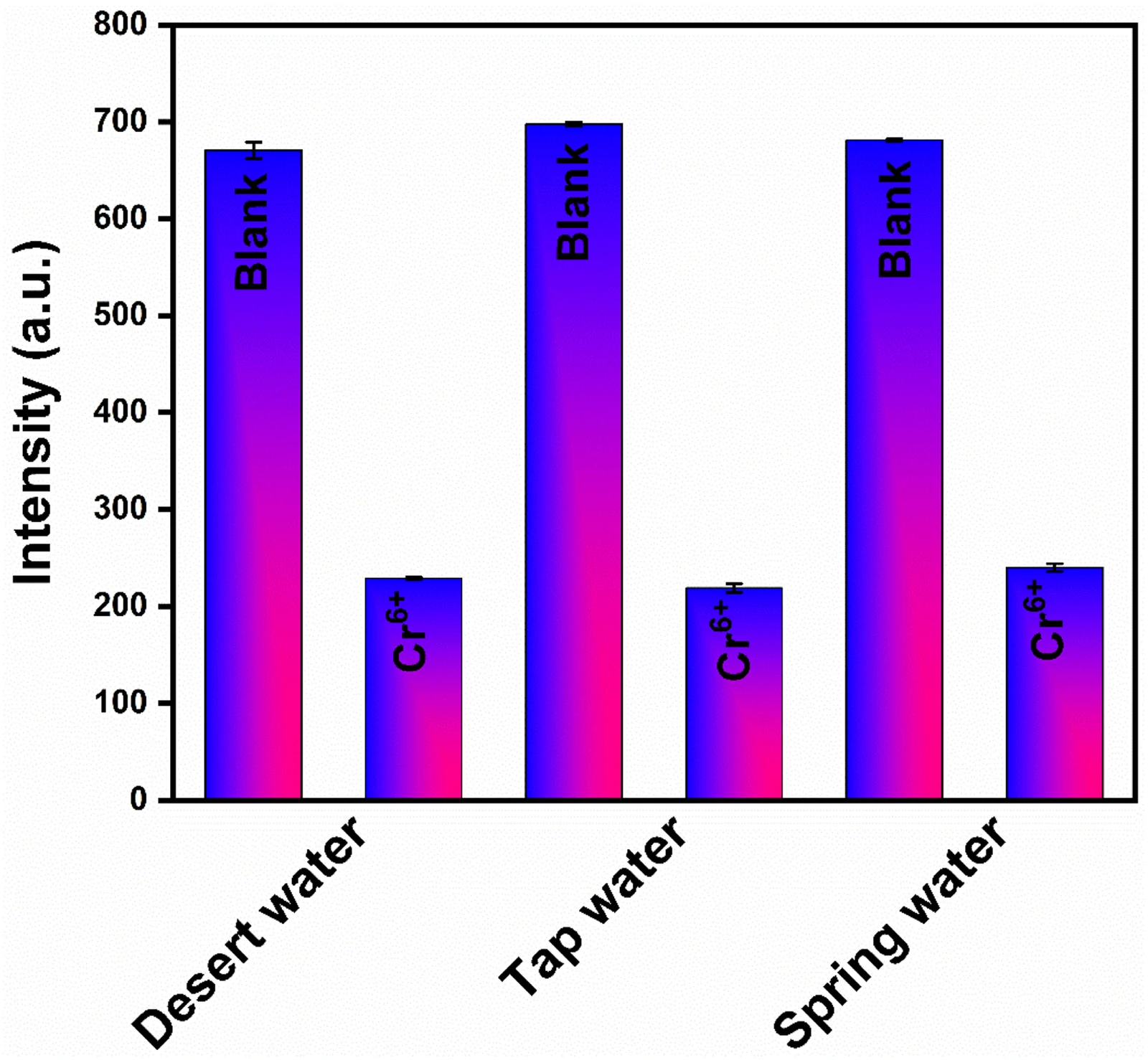
Detection of Cr^6+^ in Different Water Samples.

The sensor performance of CQD@ZIF-67@MIL-125(Ti) for Cr^6+^ detection was analysed for water samples collected from different sources. Tap water was taken from the tap located in the Biochemistry Laboratory of Kutahya Dumlupınar University. A commercially available bottled spring water from Turkey was used. Desert water was obtained from the desert region of Algeria. Water samples were passed through 0.45 µm membrane filters. Subsequently, the fluorescence intensity of CQD@ZIF-67@MIL-125(Ti) and CQD@ZIF-67@MIL-125(Ti) with added Cr^6+^ was examined. Fig. 11 shows the results obtained, and it was found that the fluorescence intensity of the water samples with added Cr^6+^ was extinguished independently of the source. Fig. 11 demonstrates that CQD@ZIF-67@MIL-125(Ti) exhibits consistent results in different real water samples and revealing its practical applicability.

## 4. Conclusion

The MOF@MOF hybrid was prepared using *in situ* modification techniques. ZIF-67 s were grown onto MIL-125(Ti) nanodisks, and a ZIF-67@MIL-125-(Ti) composite was produced. Subsequently, CQDs synthesized from banana peels were doped onto the MOF-on-MOF structure, and a CQD@ZIF-67@MIL-125(Ti) composite was synthesized. This material synthesis is reported for the first time in the literature. The synthesized composite was characterized by XRD, FE-SEM, TEM, FTIR, and FL spectrum analyses. TEM and FE-SEM results revealed a MOF@MOF structure. The photocatalytic activity of CQD@ZIF-67@MIL-125(Ti) against MB was tested under sunlight, and the photodegradation rate was found to be 91.63% after 150 minutes. The antibacterial and antibiofilm activity of CQD@ZIF-67@MIL-125(Ti) was investigated against *E. coli* and *S. aureus* bacteria. MIC values for *E. coli* and *S. aureus* were found to be 500 µg mL^−1^ and 125 µg mL^−1^, respectively. The biofilm formation inhibition rate of CQD@ZIF-67@MIL-125(Ti) for *E. coli* and *S. aureus* was calculated as 94.62% and 92.05%, respectively. The anticancer activity of CQD@ZIF-67@MIL-125(Ti) against HepG2 and HT-29 cancer cell lines has been investigated. For the HepG2 cancer line, IC_50_ values ​​at 24, 48, and 72 hours were found to be 14.35, 19.35, and 28.81 µg mL^−1^, respectively. For the HT-29 cancer line, IC_50_ values at 24, 48, and 72 hours were found to be 3.47, 7.28, and 1.22 µg mL^−1^, respectively. Fluorescence sensor experiments were conducted to determine the sensor effectiveness of CQD@ZIF-67@MIL-125(Ti) for the detection of Cr^6+^, and the results show that CQD@ZIF-67@MIL-125(Ti) is successful in detecting Cr^6+^. The LOD value for Cr^6+^ detection of CQD@ZIF-67@MIL-125(Ti) was calculated as 3.28 µM. The potential of CQD@ZIF-67@MIL-125(Ti) for fluorescent sensor, photocatalytic, anticancer, and antibacterial applications has been investigated. It is believed that CQD@ZIF-67@MIL-125(Ti) can be successfully integrated into different application areas.

## Author contribution

A. A. conceptualization, methodology, data curation, validation, investigation, writing–original draft, writing–review & editing; D. I. methodology, validation, investigation, writing–original draft; M. K. G. methodology, writing–original draft; G. K. methodology, writing–original draft; E. A. methodology, writing–original draft; F. S. conceptualization, validation, writing–review & editing.

## Conflicts of interest

There are no conflicts to declare.

## Supplementary Material

RA-OLF-D6RA05669E-s001

## Data Availability

The data that support the findings of this study are available from the corresponding author upon reasonable request. Supplementary information (SI) is available. See DOI: https://doi.org/10.1039/d6ra05669e.
