# Supplementary material for: Fabrication of in situ self-assembled MOF-on-MOF heterostructures and incorporation of biosynthesized CQD nanomaterial
Source: RSC Adv. 2026 Sep 7;16(43):53080–92. doi: 10.1039/d6ra05669e (PMC13548194; doi:10.1039/d6ra05669e)
Supplement: Supplementary file 1 [file RA-016-D6RA05669E-s001.pdf]

Supplementary Information (SI) for RSC Advances.
This journal is © The Royal Society of Chemistry 2026

Fabrication of in situ self-assembled MOF-on-MOF heterostructures and incorporation of biosynthesized CQD nanomaterial

Aysenur Aygun[1], Damla Ikballi[1], Meliha Koldemir Gunduz[2], Güllü Kaymak[3], Elif Aydın[4], Fatih Sen[1]*

[1]Department of Biochemistry, Sen Research Group, Kutahya Dumlupinar University, 43000, Kutahya, Turkiye

[2]Department of Engineering Basic Sciences, Faculty of Engineering and Natural Sciences, Kutahya Health Sciences University, Kutahya, Turkiye

[3]Training and Research Center, Kutahya Health Sciences University, 43100, Kutahya, Turkiye

[4]Department of Medical Microbiology, Faculty of Medicine, Agrı İbrahim Çeçen University, Agrı, Turkiye

***Corresponding author's email:** fatihsen1980@gmail.com*

S1. Material and Methods

Antibacterial Studies

First, bacteria were inoculated onto tryptone soy agar (TSA, HiMedia, India) and incubated at 37 °C for 18-24 hours. The composites were homogenized in sterile deionized water at a concentration of 10 mg/ml in an ultrasonic bath. Then, composites with different concentrations were prepared using this stock solution. To investigate the antibacterial properties of the resulting composites against bacteria, minimum inhibitory concentrations (MICs) were determined. The MIC value was determined by the serial dilution method. First, 100 µL of Luria Bertani Broth (LB, HiMedia, India) medium was placed in 96-well plates, and 100 µL of composite materials at different concentrations (1-0.002 mg/ml) were added. Then, 100 µL of 0.5 McFarland bacterial suspension was added to each well. Wells were prepared containing a single bacterium as a positive control and a single culture medium as a negative control. MIC is the lowest concentration visible to the naked eye that inhibits bacterial growth [1].

Optical Density (OD) measurements were performed at specific time intervals to determine the activity of the synthesized composites on the kinetic growth curve of S. aureus and E. coli bacteria. A 0.5 McFarland bacterial suspension was prepared, and then the synthesized materials were placed in 96-well plates at concentrations between 1-0.002 mg/ml. The cultures

were incubated at 37°C for 18-24 hours and measured at 600 nm (OD600) every 3 hours using a spectrophotometer (Thermo Scientific Multiskan FC) [2].

Preventing biofilm formation: The effect of the synthesized composites on biofilm formation of S. aureus ATCC 29213 and E. coli ATCC 25922 was determined by microdilution method [3]. LB broth medium containing 1% glucose was added to all wells in 100 µL amounts. After adding composites at different concentrations (1-0.002 mg/ml), 100 µL of 0.5 McFarland bacterial suspension was inoculated into each plate. A positive control containing a single bacterium and a negative control containing only culture medium were used. After incubation at 37°C for 18-24 hours, the medium was carefully aspirated, and the resulting biofilms were washed three times with 200 µL of sterile PBS solution to eliminate floating bacteria. Next, staining was performed with 200 µL of 0.1% crystal violet for 30 minutes. Any excess stain was washed off with PBS solution, and the plates were left to dry for a while. In each well, the dye that binds to the biofilm was dissolved for 10 minutes using 200 µL of 33% glacial acetic acid for gram (+) bacteria and 95% EtOH for gram (-) bacteria, and the absorbance value was measured as 600 nm (Thermo Scientific Multiskan FC). The percentage of biofilm formation was calculated as follows [4], [5].

$$\% \text{ Biofilm formation} = (\text{treated biofilm OD600}/\text{control biofilm OD600}) \times 100$$

Elimination of biofilm formation: The activity of the synthesized composites on biofilm formation of E. coli ATCC 25922 and S. aureus ATCC 29213 was investigated using the microdilution method. 100 µL of LB broth containing 1% glucose was added to all wells. All plates were inoculated with 100 µL of E. coli ATCC 25922 and S. aureus ATCC 29213 strains in 0.5 McFarland bacterial suspension. A well containing a single bacterium was used as the positive control, and a well containing a single culture medium was used as the negative control. The wells were incubated at 37°C for 18-24 hours to allow biofilm development. This resulting culture was then carefully washed once with sterile PBS. Subsequently, 200 µL of composites at varying concentrations between 1-0.002 mg/ml were added to these biofilms and incubated again at 37°C for 18-24 hours. After incubating the composites, the adhering cells were washed three times with sterile PBS solution to remove them and then stained with 200 µL of 0.1% crystal violet for 30 minutes. After washing off any remaining excess stain with PBS solution, the plates were left to dry at room temperature for a while. The dye adhering to the biofilm in each well was dissolved for 10 minutes using 200 µL of 33% glacial acetic acid for gram (+) bacteria and 95% EtOH for gram (-) bacteria, and the absorbance value was measured as 600

nm (Thermo Scientific Multiskan FC). The percentage of biofilm formation was calculated as follows [6].

% Destruction of biofilm = (1-(OD_{600} of treated biofilm/OD_{600} of control biofilm)) x 100.

Anticancer Studies

HepG2 (ATCC® HB-8065™) hepatocellular cancer cell line and HT29 (ATCC® HTB-38™) colorectal cancer cell line were commercially purchased for use in the study. DMEM (Dulbecco's modified Eagle's medium) medium + 10% FBS medium was prepared for cell culture studies in a sterile environment and added to the cells. The flasks were then incubated in an incubator with 5% CO2 at 37°C. HepG2 and HT29 cancer cells were coated with composite materials at concentrations of 250 µg/ml, 100 µg/ml, 50 µg/ml, 25 µg/ml, 10 µg/ml, 5 µg/ml, 2.5 µg/ml, and 1 µg/ml, and the cells were incubated for 48 hours. Control cells were placed only in culture medium. The percentage of viable cells in the cell population was determined colorimetrically using the MTT [3-(4,5-dimethyldiazol-2-yl)-2,5 diphenyl Tetrazolium Bromide] method. Cells grown in flasks were inoculated into 96-well microplates at a concentration of 10,000 cells/200 µL of medium 24 hours prior to application. Different amounts of composite materials were applied to the cells. The IC50 was calculated using GraphPad Prism 5.0 software. The viability of the cells after material application was compared to that of untreated control cells. The viability of untreated cells was assumed to be 100%. The cell viability percentage was calculated using the formula: "% viability rate: (Treated cells / Untreated cells) x 100" [7].

The mean ± standard deviation values of the IC_{50} results are shown. IC_{50} results were calculated as the average of three different experiments. The obtained data were analyzed using Graphpad software (Graphpad 7.0, GraphPad Software, Inc., San Diego, CA) with the help of ANOVA. $P < 0.001$ values were accepted for the results.

Photocatalytic Activity Studies

Potential applications of synthesized composite materials for dye removal from wastewater were determined using photocatalysis. The photocatalytic activity of the synthesized nanomaterials was investigated for the removal of MB dye. For a typical photocatalysis experiment, 10 mg of composite material was added to 100 ml of water and incubated in an ultrasonic bath for 30 minutes. Subsequently, 1 mg of MB was added, and the resulting mixture was left to stir in the dark for 30 minutes to ensure proper absorption balance and homogeneity. After 30 minutes, 3 ml of the solution was taken, centrifuged, and the UV-vis measurement of

the supernatant was taken. The initial measurement was considered to be CO. The samples were then exposed to sunlight, and UV-Vis measurements were taken at 30-minute intervals. [8]. The CQD@ZIF-67@MIL-125(Ti) composite was analysed under a 400 W halogen lamp (approximately 50 cm between the light source and the sample). Finally, the percentage of photocatalytic degradation was calculated using the following equations:

Photocatalytic Degradation (%): $(C_0\text{-}C_t/C_0) \times 100$

Here, C_0 and Ct are the initial and minute absorbances of MB after sun exposure, respectively.

S2. Results and Discussion

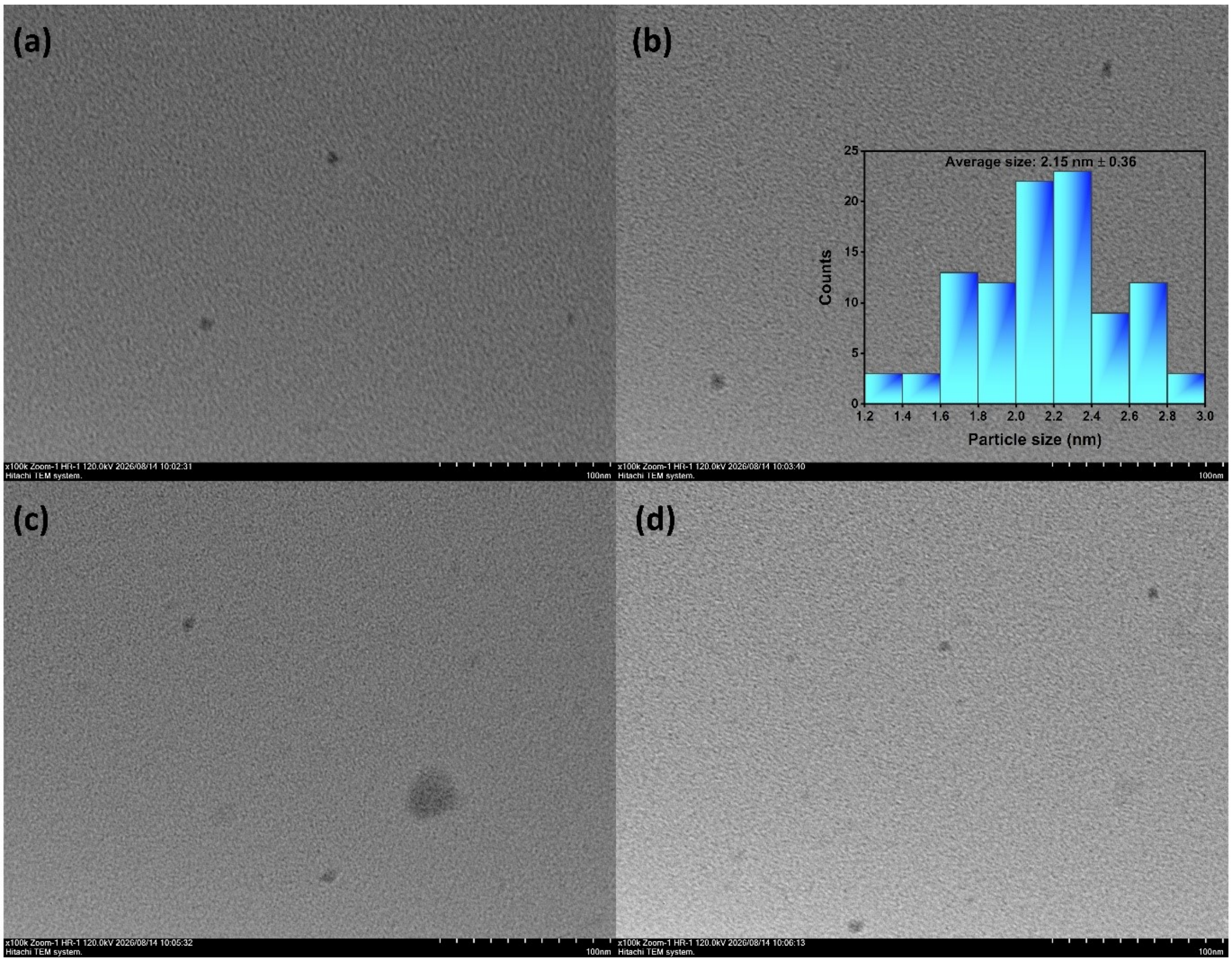

Figure S1(a-d). TEM image of CQDs (scale bar: 100 nm).

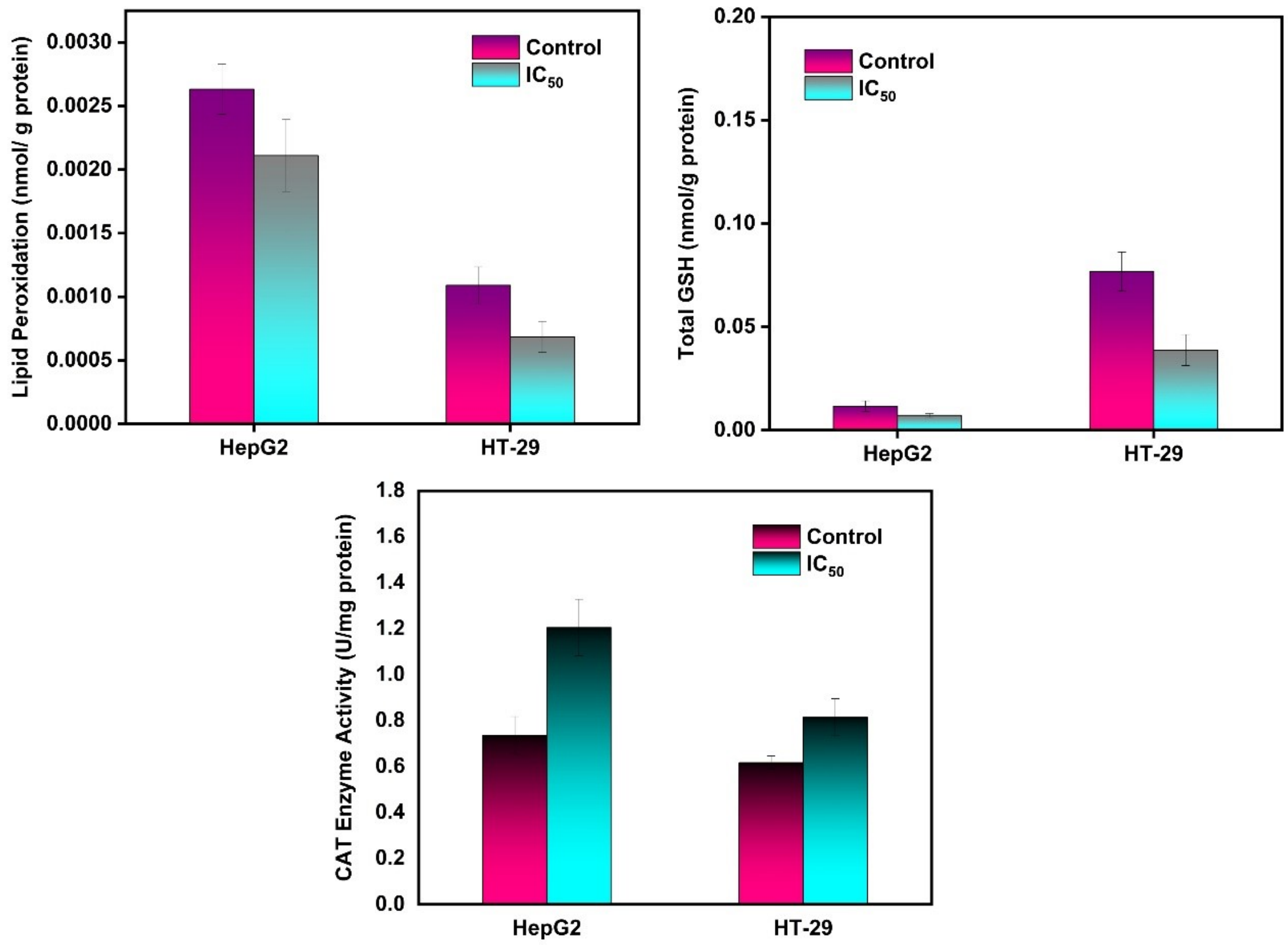

Figure S2. Oxidative stress parameters in HepG2 and HT-29 cells after composite material exposure. (Results are given as mean ± standard error.)

References

[1] L. Barnes V, D. M. Heithoff, S. P. Mahan, J. K. House, and M. J. Mahan, "Antimicrobial susceptibility testing to evaluate minimum inhibitory concentration values of clinically relevant antibiotics," *STAR Protoc.*, vol. 4, no. 3, p. 102512, Sep. 2023, doi: 10.1016/J.XPRO.2023.102512.

[2] A. Masri, D. M. Brown, D. G. E. Smith, V. Stone, and H. J. Johnston, "Comparison of In Vitro Approaches to Assess the Antibacterial Effects of Nanomaterials," *J. Funct. Biomater.*, vol. 13, no. 4, p. 255, Dec. 2022, doi: 10.3390/JFB13040255/S1.

[3] H. J. Tang, C. C. Chen, W. C. Ko, W. L. Yu, S. R. Chiang, and Y. C. Chuang, "In vitro efficacy of antimicrobial agents against high-inoculum or biofilm-embedded meticillin-resistant Staphylococcus aureus with vancomycin minimal inhibitory concentrations equal to 2 μg/mL (VA2-MRSA)," *Int. J. Antimicrob. Agents*, vol. 38, no. 1, pp. 46–51, Jul. 2011, doi: 10.1016/J.IJANTIMICAG.2011.02.013.

[4] A. K. Mishra, R. P. Thakare, B. G. Santani, S. M. Yabaji, S. K. Dixit, and K. K. Srivastava, "Unlocking the enigma of phenotypic drug tolerance: Mechanisms and emerging therapeutic strategies," *Biochimie*, vol. 220, pp. 67–83, May 2024, doi: 10.1016/J.BIOCHI.2023.12.009.

[5] P. Nguyen Trang, T. Thi Anh Ngoc, Y. Masuda, K. I. Hohjoh, and T. Miyamoto, "Biofilm Formation From Listeria monocytogenes Isolated From Pangasius Fish-processing Plants," *J. Food Prot.*, vol. 86, no. 3, p. 100044, Mar. 2023, doi: 10.1016/J.JFP.2023.100044.

[6] Q. Chen *et al.*, "Bactericidal and biofilm eradication efficacy of a fluorinated benzimidazole derivative, TFBZ, against methicillin-resistant Staphylococcus aureus," *Front. Pharmacol.*, vol. 15, 2024, doi: 10.3389/FPHAR.2024.1342821/FULL.

[7] Y. Huang, Y. Kang, A. El-kott, A. E. Ahmed, A. Khames, and M. A. Zein, "Decorated Cu NPs on Lignin coated magnetic nanoparticles: Its performance in the reduction of nitroarenes and investigation of its anticancer activity in A549 lung cancer cells," *Arabian Journal of Chemistry*, 2021, doi: 10.1016/j.arabjc.2021.103299.

[8] Y. Wu *et al.*, "Hydrogen generation from methanolysis of sodium borohydride using waste coffee oil modified zinc oxide nanoparticles and their photocatalytic activities," *Int. J. Hydrogen Energy*, vol. 48, no. 17, pp. 6613–6623, Feb. 2023, doi: 10.1016/J.IJHYDENE.2022.04.177.
